# Supplementary material for: Changes in plantar load distribution in legally blind subjects
Source: PLoS One. 2021 Apr 15;16(4):e0249467. doi: 10.1371/journal.pone.0249467 (PMC8049300; doi:10.1371/journal.pone.0249467)
Supplement: Attachment — Submitted filename: PONE.docx [file pone.0249467.s002.docx]

Thank you for the opportunity to review the manuscript titled "CHANGES IN PLANTAR LOAD DISTRIBUTION IN VISUALLY-IMPAIRED SUBJECTS" (PONE-D-20-33643). This is an interesting descriptive study on the consequences of visual deprivation on balance and plantar pressures.

The authors provide a brief but interesting introduction, but do not include all the current scientific evidence on the subject.

Below, I suggest a series of changes to improve the quality of the manuscript, which I beg you to take as constructive criticism:

1. It is easier to make changes and indicate specific errors if the text has numbered lines. It is a suggestion for future submits.

2. In the abstract, it is no longer clear from the beginning whether we are talking about "visually impairment subjects" or "blind people", since as the authors indicate at the end of the discussion, "blind subjects" is not equal to "visual impairment ". It is better to use the same word mark throughout the text.

3. There are recent studies on plantar pressure and balance in healthy subjects that may help to support certain results of this study. For example:

Sánchez-González, María Carmen et al. Visual Binocular Disorders and Their Relationship with Baropodometric Parameters: A Cross-Association Study. BioMed Research International Volume 2020, Article ID 6834591, 9 pages. DOI: 10.1155 / 2020/6834591. It would be nice to include recent articles like this to improve the introduction and discussion.

4. In the methodology, the selection criteria of the sample have not been described and the characteristics of the sample are not exactly detailed until Table 1 and 2. Where is the sample collected from? It is too striking that the sample is so homogeneous in relation to age and body mass index.

5. It talks about the two situations in which the "visually impairment" subjects are measured: with eyes open and closed, but, if they were blind, as you say later, why have these two situations been taken into account?

6. Improve the quality of Figure 1, it looks a little blurry. It would be interesting to explain what the different zones consist of in the same text and not in the figure legends.

7. It is also striking that the sample is divided almost in half into "acquired" and "congenital" visually impairment subjects. Was it done randomly or was it sampled of some kind to select them?

8. The ages at which vision was lost in the "acquired" group are very different (30 years vs 2 years), could this not affect the previous visual experience and therefore the results?

9. Figure 2, like Figure 1, should be explained in text to reduce the figure legend.

10. Could tables 6 and 7 be unified so that the results of P mean and P max can be equated?

11. The discussion must be improved, including references that can justify the results, as well as not generalizing the results (as in the first section) where it literally says "the visually impaired participants had higher pressure in the first and second metatarsals". This has not been the case in all cases, it would be necessary to specify and try to justify these findings. Be careful with generalizing as the sample is not too large.

12. Merge the references in the discussion.

13. The last paragraph that explains the blindness, should be explained much earlier in the text of the manuscript, including putting it in the sample and its selection.
